# System-level brain modeling

**DOI:** 10.3389/fncom.2025.1607239

**Published:** 2025-07-16

**Authors:** Birger Johansson, Trond A. Tjøstheim, Christian Balkenius

**Affiliations:** Lund University Cognitive Science, Department of Philosophy, Lund University, Lund, Sweden

**Keywords:** computational brain models, system-level, real-time, robotics, modeling

## Abstract

System-level brain modeling is a powerful method for building computational models of the brain and allows biologically motivated models to produce measurable behavior that can be tested against empirical data. System-level brain models occupy an intermediate position between detailed neuronal circuit models and abstract cognitive models. They are distinguished by their structural and functional resemblance to the brain, while also allowing for thorough testing and evaluation. In designing system-level brain models, several questions need to be addressed. What are the components of the system? At what level should these components be modeled? How are the components connected—that is, what is the structure of the system? What is the function of each component? What kind of information flows between the components, and how is that information coded? We mainly address models of cognitive abilities or subsystems that produce measurable behavior rather than models that to reproduce internal states, signals or activation patterns. In this method paper, we argue that system-level modeling is an excellent method for addressing complex cognitive and behavioral phenomena.

## 1 Introduction

System-level brain models are biologically inspired computational frameworks that aim to replicate selected aspects of brain function from a systems-level perspective—that is, as a collection of interconnected components with specific computational properties and interactions (Balkenius et al., 2020, 2010). These models span a spectrum, from networks that emulate processing in defined brain regions to architectures composed of computational modules with only a loose resemblance to their biological counterparts. In either case, there is a structural correspondence to the biological brain, as individual components of the model are designed to represent specific brain regions rather than abstract cognitive functions, as is common in many cognitive architectures.

In this methods paper, we present key aspects of brain modeling using a system-level approach. We address practical questions that modelers should consider during the construction of brain models and offer insights into model validation.

Systems approaches to design and modeling are well established in engineering and industrial applications. However, such methodologies have not yet seen widespread adoption within computational cognitive science or cognitive neuroscience, despite foundational philosophical work dating back to the 1970s (Laszlo, 2021). Nonetheless, systems-level modeling offers distinct advantages for brain modeling, particularly in terms of compositionality and hierarchical organization. Compositionality allows for the integration of smaller, self-contained models into larger systems in a modular, plug-and-play fashion. Hierarchical organization, in turn, enables modelers to abstract complexity by embedding subsystems within higher-level structures, facilitating scalable and comprehensible model architectures.

System-level brain modeling is a systematic approach that develops brain models by successive refinement using structural and functional validation. It aims at reproducing behavioral data and the main benchmark of a system-level model is to what extent it reproduces experimental behavioral results in simulation or in controlling a robot.

A system-level brain model suggests specific functional roles of different brain regions without necessarily dwelling on the detailed neuronal processes in those regions. This approach provides some distinct benefits and challenges compared to approaches that do not employ systems-thinking or that focus on isolated functions and networks rather than the brain as a complete and integrated system. This topic will be given a full treatment below, after we have looked at modeling in a broader context.

There are several motivations for constructing brain models, which can broadly be categorized into two main aims. The first is to enhance our understanding of actual brain processes (Farrell and Lewandowsky, 2018). Here, models are often intended to address fundamental questions about cognitive phenomena such as learning, memory, attention, and motor control by attempting to replicate underlying neural mechanisms. Additionally, system-level models serve as valuable tools for theorizing about the neural bases of psychiatric and neurological disorders.

The second aim is to develop neuromimetic or biomimetic systems (Vincent and Mann, 2002), where the objective is to design technical systems that emulate certain properties of the biological brain. Since the brain is known to solve complex problems efficiently, it is natural to draw inspiration from its architecture and processes to inform the design of artificial systems. In the following sections, we focus specifically on the modeling of cognitive and neural processes.

## 2 Computational models

A model can serve as a valuable instrument for verifying the accuracy of our understanding, particularly when dealing with complex systems. By analyzing the model's behavior and outcomes, we can ensure that the underlying principles and assumptions align with our expectations and that the model accurately represents the system in question. Furthermore, by exploring the model parameters and by providing a wide range of input data yields a more comprehensive understanding of the model's inner workings (Farrell and Lewandowsky, 2018).

Regardless of type, a brain model is always a simplification. It reproduces some aspects of the real brain while intentionally disregarding others. It is indeed the case that “the best material model for a cat is another, or preferably the same cat” as suggested by Rosenblueth and Wiener (1945), but a model without simplification typically does not contribute to our understanding. Instead, we need to make informed decisions about what aspects of the real brain to keep in the model and which to keep out.

The rationale for such decisions depends on the model's intended purpose. Typically, this involves producing a measurable output, whether in the form of internal neural signals or observable behavior. If the goal is scientific understanding, then the model output should be compared to corresponding outputs from empirical studies of the brain. Conversely, in engineering applications, the model's performance should align with specific technical requirements.

It is important to acknowledge that all models are to some extent imperfect except possibly in very simple cases. As stated by Box (1976), “All models are wrong, some are useful.” Thus, modeling should be viewed as an iterative process of approximation, with models continuously updated to incorporate new data. Evaluating a model involves assessing both how well it reproduces known phenomena and the extent to which it can generate testable predictions.

Crucially, a model must be capable of generating predictions that could potentially be incorrect, and when they are, it is necessary to have criteria for deciding whether it is because the model is incomplete or simply wrong. This is often very challenging to determine and puts a light on the need to have a clear scope for the model. The model should make correct predictions within that predefined scope, but may be inaccurate when tested outside it.

Ideally, the predictions made by a model should be used to devise new experiments that can confirm or falsify the model and lead to a revised model that can hopefully explain the new conflicting data. However, this is seldom achieved in practice. There are two main reasons for this. First, the researchers designing computational models are often not the ones doing the experiments. They may not even communicate with them, possibly because they have different goals in their research and different understanding of what is important in research. Second, the time scales of modeling and experimental work are very different. A model can be changed in an instant, but experimental work usually take months or even years. Even if the experimenters try to test predictions made by a model, the modelers cannot realistically wait for years before refining their model.

An alternative approach involves ensuring that a model accounts for as much existing empirical data as possible while relying on a minimal set of assumptions. Furthermore, the assumptions made should as much as possible be motivated by other factors than the data it is trying to reproduce. There is a critical difference between a model *of* the data, and a model of the *process* that generated the data. The former is usually approached by standard statistical modeling techniques while the latter requires some form of a process model.

### 2.1 Models, parameters and predictions

Parameters can be categorized into two main types: free parameters and fixed parameters (Kline, 2023). Free parameters are estimated from the data, with the goal of ensuring that the model accurately fits the data. On the other hand, fixed parameters are not derived from the data set; rather, they are predetermined constants that provide meaningful information to the model. For instance, fixed parameters can include the number of neurons in an artificial neural network (ANN) layer. While the number of free parameters is often a primary concern for modelers due to their direct impact on model performance, fixed parameters generally receive less attention, as they are meant to establish the foundation of the model without influencing adaptability based on input data (Farrell and Lewandowsky, 2018). Note, however, that there are cases where fixed parameters, such as network size are optimized for best performance, for example, in many deep-learning models.

We can further distinguish between the following types of parameters: **Fundamental constants** are parameters that are not a function of any other constants, such as Avogadro's number. They are also to reflect some aspect of physical reality such as the gravitational constant, *G*. **Empirical constants** are parameters that are set to reflect an empirically measured value such as the resting potential of a neuron or the velocity of an action potential through the axon. **Scaling parameters** scale the input or output of model to measured magnitudes or to real time. These parameters do not influence the processing of predictions of the model in any other way and only serve to map values from one range to another, for example, between arbitrary values and standard units. **Metaparameters** are sometimes also called hyperparameters and are values that control the processing in the model such as the learning rate. **Weights** are the free parameters that change during the simulation of the model. In brain models and artificial neural networks, these are typically synaptic weight or connection weights. Sometimes, these are called just “parameters.” Myung and Pitt (1997) identified three factors that determine a model's flexibility, with the number of free parameters being the first factor. They asserted that a model with a greater number of free parameters would fit the data better than a model with fewer parameters. However, it is essential to consider the trade-off between model complexity and generality. While fewer parameters can increase the generality of the model, allowing it to be more applicable to a wider range of scenarios, using too few parameters may lead to an opposite effect, decreasing the model's generality. Therefore, striking a balance between the number of free parameters and the model's generality is crucial for achieving optimal performance in various applications. When the number of parameters increases, so does the risk of overfitting. Is the behavior of the model the result of the actual model or the large degrees of freedom introduced by the free parameters? In the words of Stanislaw Ulam, “Give me 15 parameters and I can make an elephant; give me 16 and I can make it dance” (Weiss et al., 2003). In statistics there are methods such as the Akaike information criterion that weigh the predictive ability of a model against its complexity (Akaike, 1973, 1974). Brain models usually afford no such luxury. Instead it is the good judgment of the modeler that must determine how complex the model must be.

In general, there should also be as few degrees of freedom as possible. This means minimizing the number of parameters as much as possible and if possible motivate the parameters independently of the data that the model sets out to explain. This is a well-know problem with many artificial neural network models that have a very large number of parameters and thus can be trained to do almost anything. Although a network like this can be useful as a technical tool, it is not necessarily very useful as a model of a biological brain. Thus, connectionist neural network models may offer limited explanatory power (but see Whittington et al., 2021 for an intriguing example of how transformer architectures used for large language models have commonalities with a model of the hippocampus).

When testing a model, the number of predictions should be maximized. This means that the model should ideally be tested on all data that is available within the explanatory scope of the model.

The evaluation of a model can be either qualitative or quantitative. Qualitative evaluation often takes the form of inequalities, such as checking whether the output of the model is higher for one condition than another without looking at the actual value. This is especially useful in models where the output is not precisely scaled to empirical data and also have the advantage that it limits the number of parameters that need to be used. Quantitative evaluation can either use direct comparison with empirical data using some form of distance metric or a full statistical comparison of the model output and the dataset.

**Figure 1:**
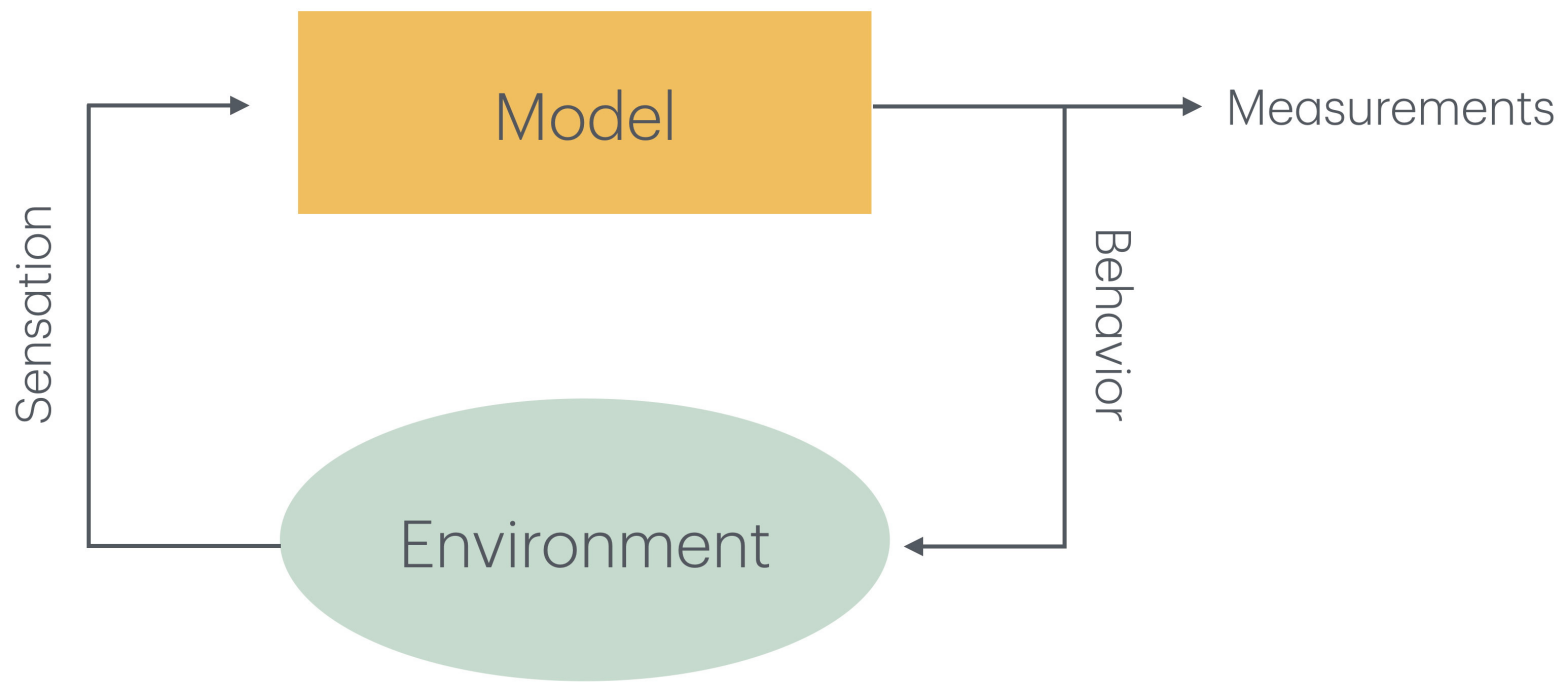
The model produces measurable behavior that also influences the environment that can either be simulated together with the model or consists of the real world influenced by the actions of a robot. The state of the environment changes as the result of the behavior or the model and produces new sensory input to the mode.

In either case, it is essential that the output produced by the model can be compared to measured behavior (Wilson and Collins, 2019). This may entail putting the model inside a simulated agent that can act in a simulated environment. This is also true in cases where a brain model is used to control the actions of a robot (Figure 1). In this case, it is usually not possible to expect perfect scaling to empirical data, but qualitative measures are still possible.

It is also critical that the predictions of the model can be experimentally tested in new experiments. As new data becomes available, it can be compared to the predictions made by the model. We have to accept the fact that this data is mostly not a result of predictions made by the model but motivated by other factors. Although experimental data are often collected independently of model-derived predictions, they can still serve as valuable validation points when assessing model robustness.

However, it is important to remember that not all published results are correct (Ioannidis, 2005; Open Science Collaboration, 2015). This is a consequence of the empirical method where there is always a probability that an incorrect results was obtained by chance. It is therefore of essence to take care to avoid overfitting the model to data. In fact, one very useful aspect of models is that they can suggest what experimental results need further scrutiny (Cf. Barry, 2006). If a model is able to reproduce a large number of experimental results except one, then there is reason to try to empirically reproduce the results of that experiment. Similarly, if additional assumptions are needed just to account for a single phenomenon, then the model is likely to suffer from overfitting. We do not claim that a model on its own can invalidate empirical results. However, it can point out what results do not fit within the framework of the model. Experimental work is always necessary to determine whether the model or the experimental results are incorrect and will ideally lead to subsequent revision of the model to accommodate the data if necessary.

A successful model should be able to reproduce all (correct) data using the same parameters. It should not be necessary to adapt these for each data set unless it can be motivated independently of the data. An interesting example is the famous Rescorla-Wagner model (Rescorla, 1972) that reproduces the results of a range of classical conditioning experiment. Although the model provides some fundamental insights into the interaction between different stimuli during learning, it is necessary to change the parameters to fit the particular experimental protocol which makes the model less suitable as a general explanation of the phenomenon (Gallistel, 1990).

### 2.2 Computational soundness

Another important factor in a computation model is that it is computationally sound. One way to approach this is through David Marr's three levels (Marr, 1982). He presented a framework of how the brain processes information and three levels of analysis can be applied to understanding cognitive functions. The highest level of analysis is the computational level and concerns the goal of the cognitive system. The intermediate level is the algorithmic level that relates to the specific steps or operation that the cognitive system uses to achieve its goals. At the lowest level, the implementation level, concerns the physical implementation such as neurons and neuron circuits.

The primary contribution of this work is that it stresses that it is necessary to understand the problem that the brain is trying to solve before looking for the solution in the brain (Gallistel, 1990; Balkenius et al., 2023). What are the ecologically valid problems the brain is trying to solve, and what are their properties? As important as this question is, it is not always easy to answer. It is also easy to design a model that does not in fact take the physical properties of reality into account. Even Marr himself is guilty of this when he proposed a model of visual depth processing that assumes that all surfaces have a fixed orientation relative to the eye, which is obviously not the case in nature (Marr and Poggio, 1979).

Numerous research paradigms have demonstrated a tendency for findings to align closely with researchers' initial hypotheses. When conditioning is thought to be behind all control of behavior then we find the brain is filled with associative mechanisms. When cognition is thought to be controlled by different modules with names such as memory, attention and motor control, those become the boxes found in the brain. This in turn carries over to the models that are built that will contain exactly those components.

This exemplifies the risk of confirmation bias, wherein expectations may inadvertently shape both experimental design and interpretation and is something to be cautious about when designing system-level brain models. It is necessary to be very aware of the actual scope of the model and to decide on an appropriate level of abstraction.

## 3 System-level brain modeling

System-level brain models represent a specific class of network models that emphasize the computational roles of each of the including brain regions, and treat the brain as a functional network of interconnected components that produces a measurable behavioral output.

System-level modeling is used in various fields to analyze complex systems such as airplanes, space rockets, biological processes and financial systems. For these complex systems, predicting the impact of component modifications on overall system performance can be challenging. By using system-level modeling, the focus is on the complete behavior of the system rather than the details of its components. Each component in the system is modular and together with all other components make up the system as a whole.

Using a system-level modeling approach for the human brain, refers the process of creating a computational model of the brain that simulates the overall patterns of neural activity in different brain regions. This representation of the activity in the neural networks and their interaction in the brain can shed light on how the brain processes information and generates behavior. Adopting a system-level approach can also be highly effective for developing robotic behaviors inspired by the human brain.

System-level brain modeling is ideal for simulating or analyzing systems composed of multiple interacting subsystems, such as in robotics. For instance, in robotics, this approach can integrate sensory inputs, motor controls, and decision-making algorithms to create a cohesive and functional robot.

Furthermore, system-level brain modeling employs a modular architecture, wherein distinct components are responsible for specific functions. This modularity facilitates the testing of various submodels against one another. For example, the behavior of a robot can be assessed by interchanging different cerebellum models. This approach enhances the ability to evaluate and compare the performance of diverse brain models within the context of the entire system.

The goal of system-level brain modeling is to create a comprehensive model of the brain that can be used to study various aspects of brain function, such as perception, attention, executive functions or reflexes. These models can be used to test hypotheses about the human brain, predict the effects of brain damage or disease, and design and optimize brain-machine interfaces or as control system in robots. It should be emphasized that the functions under investigation arise from the interactions of the different components in the model without there being any one-to-one correspondence between components and functions. For example, “attention” may be the result of interaction between a large number of computational components, none of which can be labeled “attention.” Developing a system-level model of the human brain remains a significant challenge. In engineering, a system-level model can increase the understanding of a complex system such as an aircraft as the model can help identify potential issues, and design optimization that meets performance and safety criteria, resulting in enhanced aircraft performance, safety, and efficiency. In these complex systems, both component details and predicted system behavior are known. Completeness is a major difference between engineering system-level models and models of the human brain. Our comprehension of the brain's inner workings is far from complete, particularly in modeling how different brain regions influence each other.

Other challenges include modeling the intricate interactions between various subsystems, such as neural networks, sensory inputs, and motor outputs, which are highly complex. Modeling the dynamic and non-linear nature of these interactions requires advanced algorithms and computational methods.

Integrating data from different sources and scales (e.g., molecular, cellular, and system-levels) is challenging. Ensuring that the data is compatible and accurately represents the biological processes is crucial for the model's validity.

To be able to create a system-level model of the brain, there are a number of questions that need to be considered (Balkenius et al., 2010):

**What are the components of the system?** In system-level brain modeling, the components are typically represented as a network of interconnected nodes. The relationships between these components are important for simulating the behavior of the system. Components can be brain areas that are known to perform a specific function, such as memory coding, motor control or visual sensory processing. Using larger brain areas as components, the area is modeled as collections of neural circuits that all contribute to perform specific tasks.

Components can also be modeled at different levels of description. On the lowest level, brain models focus on single neurons (Rotstein and Nadim, 2020). In such models, the components are typically intra-cellular, like ion channels and receptors (Abbott, 1999), but could also include cellular machinery like mitochondria (Woo et al., 2021). A major steps toward more complex models of single neuronal cells was the Hodgin-Huxley model (Hodgkin and Huxley, 1952). This model is based on the electrophysiological properties of squid neurons and focuses on simulating conductances of various ions like calcium and potassium and how these electrical dynamics together produce action potentials. Single cell models can also include parts like dendrites, soma, and the axon as components (Rall, 1964; Almog and Korngreen, 2016).

At the next level, several neurons communicate using electrical and chemical signals. Using this abstraction, the neuron is simulated as receiving inputs from other individual neurons and integration these inputs, to produce outputs in the form of action potentials. Focusing on smaller circuits involving a few, or often pairs of neurons can elucidate dynamics such as rhythm generation, resonance behavior, and attractor dynamics (Brown, 1914; Kopell and Ermentrout, 2002). In such small circuits it can make sense to use different types of neurons as components, such as excitative and inhibitive neurons (Kopell and Ermentrout, 2002). Groups of neurons that are connected and influence a specific function can be modeled as a network of interconnected neurons, that is the overall activation of the neurons instead of each individual neuron. For example, Marder (2002) reports how the study of small-scale non-vertebrate neural systems contributed to changing the view of neural networks as static entities toward realization of their dynamic nature. In particular, the study of swimming in molluscs (Getting and Dekin, 1985) and the stomatogastric ganglion of lobsters and crabs (Nusbaum and Beenhakker, 2002) have shown how changes in topology, and the interaction of excitation, inhibition, and modulation contribute to producing behavior.

At the system-level, a component corresponds to a whole nucleus in the brain or to a neural field of neurons. Such network models aim to simulate the connectivity patterns observed in the brain's neural circuits, often based on data from neuroimaging techniques such as diffusion tensor imaging (DTI) or functional magnetic resonance imaging (fMRI). Such connectome-based models aims at providing insights into the large-scale organization of the brain.

Breakspear (2017) reviews models of large-scale brain activity based on dynamical systems theory, in essence presenting two types: neural mass models (NMM) and networks of such models, and neural field models (NFM).

The core principle of NMMs is to represent the average activity of a local, interacting population of neurons as a single, aggregated entity (Freeman, 1975). Combining multiple NMMs into large-scale brain models offers a valuable approach to studying brain dynamics. Prominent examples of NMMs include the Wilson-Cowan model (Wilson and Cowan, 1972) and the Jansen-Rit model (Jansen and Rit, 1995).

NFM can be used to model both cortical and subcortical areas. For example, Roberts et al. (2016) use this framework to construct a network based on the connectome of the human brain. This network is then used to investigate the influence of spatial geometry on brain topology, allowing the authors to quantify the degree to which segregation, integration, and modularity in the brain is due to its spatial embedding. NFMs model cortical surfaces like a continuous field where activity is described by a wave function (Jirsa and Haken, 1996). NFMs have reproduced the typical wavefront dynamics that have been observed in particular in sensory- and motor cortices (Muller et al., 2014; Rubino et al., 2006).

On the highest system-level, components are purely functional. This may be necessary for a model that produces measurable behavior as output. At this level, it is possible to use Bayesian models of cognitive processes, based on the idea that neural populations in animals mediate probability distributions in part shaped by sensory sampling of the environment. This process can then be simulated by means of computer models of the same probability distributions.

As an example, Yuille and Kersten (2006) describes the process of using Bayesian inference to perform object recognition. The authors make the case that top-down expectations make object detection more robust, but requires learning of a possibly very large data set in order to extract the necessary syntax and grammar for the generation of those expectations. However, such a model might be able to cope with even very complex natural scenes by means of “analysis by synthesis;” this implies that a scene is viewed as composed of several samples from probability distributions representing concepts or categories of things. By generating suitable instances of the categories in the syntax set such that they match the bottom-up signatures of the scene in question, recognition of individual components may be achieved.

For Bayesian motor control, Kording and Wolpert (2006) argue that probabilistic approaches makes for useful models of movement under uncertainty. Although several sensor systems can contribute to the brain keeping track of a reaching hand, for example, the loss of critical ones like vision will make actions more error prone. The speed of movement will affect uncertainty as well. Actions such as reaching carry costs, most saliently in terms of metabolic energy. Deciding which exact movement to engage in from a near infinite space of possibilities can be modeled in terms of Bayesian decision theory. This framework, where decisions are happening at each point in time, is identical to optimal feedback control in control theory.

Each abstraction layer presented in the text can enhance our understanding of the human brain. However, we want to argue that the system-level is particularly useful when studying the function of the brain in various cognitive processes where it is the behavioral output that is the index of the function.

**Which function is performed by each component?** The function refers to the specific role or purpose of the brain region or circuit defined as a component. Each component performs a specific operation contributing to the system's overall function. In a system-level brain model, there are strong claims about the relation between each component in the model and the function of a specific brain region. This does not mean that the component performs a complete function on its own or that it is functionally impenetrable (See Fodor, 1983, for an opposing view). Instead, the claim is only that the operation of each component can be described in functional terms.

Hence, in single cell models, components can compute changes in ion currents, and how these currents contribute to forming action potentials (Hodgkin and Huxley, 1952). In small circuit models, the components may produce spikes, and transform excitatory and inhibitory spikes into complex rhythms at different frequencies (Bastos et al., 2018; Mejías et al., 2016). In neurocognitive models, (see e.g. O'Reilly et al., 2016) components can do both Hebbian- and error-driven learning. Inhibition also affords winner-takes-all selection mechanisms which can be found in many parts of the brain, including the basal ganglia and the medial prefrontal cortex. Connectionist models, and deep convolutional networks in particular, have become standardized tools for patterns recognition and classification, perhaps most well known in the context of images (LeCun and Bengio, 1998). In these models, components work by essentially combining various types of matrix operations, the most important of which is dot-product matrix multiplication. In Bayesian models, components perform transformations on probability distributions; a fitted model can thus yield samples from a complex combination of individual probability distributions and these samples can for example produce behavior.

**How do the components interact with each other?** In system-level brain modeling, the connectivity between brain areas or circuits involved in various behaviors represents the interactions between components. These interactions, which simulate the transfer and processing of information across different brain regions, are crucial for understanding the collaborative mechanisms underlying behavior and cognition.

Components can also be organized hierarchically, in particular when a model spans multiple descriptive levels. However, hierarchies can also be a practical method of grouping components that are the same descriptive level. For example, it is useful to follow the normal anatomical hierarchy of the brain when designing a system-level model. This is particularly useful for large-scale models where the number of interacting components may range in the hundreds. To specify the interaction between the components is essential both to understand information transfer in the model and for structural validation of the model (see below).

**What information is transmitted between the components?** In the highly interconnected human brain, the transfer of information between different regions is essential for understanding behavior, cognition, and perception. Information transfer refers to the process by which neural activity is transmitted and mediating information between structures or circuits between different brain regions.

We use the word “information” in the sense used within information theory (Shannon, 1948), that is, as a measure of the uncertainty reduction or the ability to distinguish among a set of possible messages or events. Hence, it is not necessarily tied to the meaningful content of the message interpretable to humans, but rather to the mathematical properties and probabilities associated with the transmitted data.

The question about information contents is again strongly connected to the claims of the model about how different components, and hence their corresponding parts of the brain, are able to influence each other. So, in single cell models, the information can represent the concentration of ions, or changes in ion currents, for example. In circuits of spiking units, information represents electrical voltage as a function of time, implicitly including frequency and phase information, while in connectionist models the information may be interpreted as the activity of neuronal populations in some cases, while there may be no obvious biological interpretation in others. In Bayesian models, finally, the information flowing between components may be samples from multidimensional distributions.

**How is this information coded?** Coding is the neural activity that represents different types of information or stimuli processed by the brain and is a fundamental concept as it helps to explain how the brain represents and processes information. Different codings affect how the information is processed by the brain. Although at the neuronal level, the most common intuition about information mediation is activity in terms of action potentials in the biological brain, when zooming out to consider populations, these action potentials afford a rich array of coding possibilities. When taking into account also neuro-modulation, the possibilities grow but the complexity grows with it, and can become challenging to understand and make use of.

Although coding may appear peripheral but in a system-level model, it is in fact essential to understand the processing in each component. How information is coded greatly constrains the possible architectures of the complete model as well as the modeling of the functional properties of each individual component. The choice of spiking neuron models (Rieke et al., 1999), rate coding (Van Rullen and Thorpe, 2001) or some form of population coding (Pouget et al., 2000) will greatly influence the overall structure of a system-level model. In connectionist models, where information is structured in vectors or tensors, information may be coded as real numbers in some interval, or e.g., a one-hot format for selection.

In the context of information coding it can also be useful to use symbolic systems as a contrast. Cognitive science began with symbolic models of cognition, where the brain-as-computer metaphor was the dominating one (see Kotseruba and Tsotsos, 2018 for a review of cognitive architectures, including symbolic ones). Although symbolic models can also have a structural component, its components typically have direct correspondence to different cognitive functions.

**How is timing modeled?** Most models include some aspect of time sensitivity either explicitly or implicitly in the form of time steps. When designing a model it is necessary to decide how time is handled. For a purely qualitative model, it may not be important, but when the produced output will be compared to measured signals it is necessary to know what the time scale is. For simulations, it may be enough do decide on a correspondence between a simulation step and real-time. However, for a brain model that is to control a robot, it is often necessary to use a real-time framework to implement the model.

In the context of levels of abstraction, and as mentioned above, smaller circuits involving only a few neurons are useful for testing hypotheses about time-related phenomena like rhythms, resonance, attractor dynamics and so on. Kopell and Ermentrout (2002), for instance, emphasize the role of delays in producing various rhythmic signatures; this delay can be due to characteristics of receptor proteins, but is often due to the length of axons through which signals travel. Also using crustaceans in the form of the crab *Cancer borealis*, (Nadim et al., 1998) showed how interacting oscillator networks can produce sophisticated rhythmic patterns. More specifically, they showed how neuronal oscillators outside the crab's central nervous system and associated with its digestive system together regulate digestive rhythms. Here, a rapid oscillator at the entry to the gut modulates the slower rhythm of the gastric mill. This work indicates that oscillator units can compose into larger systems to perform vital functions in organisms.

This ability of oscillators to compose dependent on tempo (i.e., frequency) and rhythm (i.e., patterns of fast and slow tempo) allows for a highly flexible way of recruiting and handing off neural units at several system-levels to form transient networks and implement functions. A key aspect of this is the ability of a coherent group of units to entrain recruited units into their specific rhythm. This principle of “communication through coherence” suggests that synchronized neural units can process and communicate information more efficiently (Fries, 2005).

Take simulation of motor control as a specific example. A group of neural units connected to motor units (i.e., muscle fibers) can entrain spinal motor neurons which again activate muscle fibers. This synchronization, known as “corticomuscular coherence” can yield more steady and powerful muscle activation compared to when neural units act in a more random, unsynchronised way (Mima and Hallett, 1999). At the same time, by changing rhythmic phase, a big coherent group of neural units can split into several distinct ones, allowing for e.g., fine motor control of fingers, a phenomenon known as “event-related desynchronization” (Pfurtscheller and da Silva, 1999). Functional changes in rhythms and phase like this is typically handled by careful control of fast inhibitory units which can modulate the phase of rhythms sufficiently so that they can run stably out of sync (Haken et al., 2004). As described in models of dynamics of coupled oscillators, systems like this tend to settle into phase (0 degrees) or antiphase (180 degrees), which allow information to be bound or segregated. From the systems perspective, neural implementations that support phase and rhythmicity may therefore produce dynamics that can be used as building blocks to compose higher order functionality—essentially providing a foundational “syntax” for neural computation (Buzsaki, 2006).

The crucial role of time can be implemented as delays on connections between components. If multiple connections with different delays are used it becomes possible to implement different forms of tapped delay lines (Desmond and Moore, 1988) and spectral timing models (Grossberg and Schmajuk, 1989). This is particularly important in the modeling of timing dependencies in classical and operant conditioning (Schmajuk, 2010).

In contrast, connectionist systems do not typically exhibit dynamical properties at all; these models are in this regard time-independent functional input-output components. The same is the case for probabilistic models that sample from distributions.

## 4 Simulation frameworks and model architecture

System-level brain modeling involves two essential components: the actual architecture of the model and the simulation framework used to run it. These two parts are often conflated in the literature, and it is not uncommon for publications to conflate model architecture with the computational tools used for implementation. This overlap is understandable, as the development of the architecture and the simulation environment is often tightly interwoven. Algorithms implemented by a framework may become integral to the architecture itself, further blurring the lines. However, it remains crucial to differentiate between these components to better understand the structure and function of system-level models.

A variety of frameworks have been developed to support the simulation of large-scale brain dynamics. The Virtual Brain (TVB) is a neuroinformatics platform designed to simulate whole-brain activity based on data from neuroimaging (Sanz Leon et al., 2013; Schirner et al., 2022). It models brain regions and their connectivity to study emergent phenomena such as oscillations and disease states. NEST (Neural Simulation Tool), while primarily known for detailed neuron-level simulations, also supports higher-level modeling of simplified neuronal populations and networks of brain regions, with a focus on system-wide dynamics rather than individual spiking activity (Diesmann and Gewaltig, 2001).

The Brain Dynamics Toolbox (Heitmann and Breakspear, 2017), a MATLAB-based framework, models the collective behavior of neural populations using differential equations. It emphasizes macroscopic brain activity over microscopic neuron-level detail. Similarly, the Human Brain Project's Brain Simulation Platform (BSP) enables simulations at the systems level, integrating structural and functional data from connectomes to model interactions between brain regions (Schirner et al., 2022).

Cedar is a framework developed to support models based on dynamic field theory (Lomp et al., 2016). It is closely tied to the building blocks of that theory and is capable of constructing complex models of interacting neural fields. Cedar has also been used in robotics (Tekülve et al., 2019), providing control mechanisms based on dynamic neural modeling.

Ikaros is another system-level modeling framework (Balkenius et al., 2010, 2020), similar in some ways to Cedar but not tied to a specific theory. It supports the development of large-scale models and allows for both simulated and real-world validation using robots (Johansson et al., 2020). Ikaros runs model components in parallel and uses a rendezvous mechanism for inter-component communication (Andrews, 1991). Both Ikaros and Cedar use the first-order Euler method to update the state of the simulated model, a method commonly used in artificial neural networks and sufficient for system-level simulations. Both platforms have been used to control robots.

Each framework presents specific advantages and limitations, and selection should be based on the research objectives and modeling requirements. In section 3, we outline a series of considerations that the modeler needs to contemplate. The answers to these questions assist in selecting the appropriate simulation tool for the intended purpose. For instance, if the focus is on achieving a highly realistic model of each neuron within the system, a tool detailed enough to capture these intricacies must be chosen. Conversely, if the goal is to model various behaviors of a robot, a higher-level approach may be preferable, where modeling occurs at the level of neural nuclei.

Across these platforms the focus is on understanding brain function as an integrated system. They employ mathematical models of neural populations and brain connectivity, rather than simulating individual neurons in fine detail.

### 4.1 Real-time aspects of brain simulators

Very few brain simulation frameworks take real-time aspects into account. Although time is often a factor in the models, there is seldom any attempt to produce precisely timed outputs. This makes sense for a pure simulation, but when brain models are to be used to control robots, this becomes increasingly important, especially for models directly involved in motor control.

Real-time systems and non-real-time systems differ primarily in their timing requirements and the consequences of not meeting those requirements (Laplante, 2004). Real-time systems have strict timing requirements and deadlines that must be met and are designed to respond to events or inputs within specific time constraints. Failing to meet timing constraints can have severe consequences. This does not mean that real-time systems necessarily need to be fast. However, they often prioritize quick response times to events or inputs and minimizes latency to provide timely outputs. To meet such timing demands, real-time systems require sophisticated scheduling and task management mechanisms.

Real-time-models make it necessary to explicitly differentiate between rate parameters that depend on time, such as a learning rate, and other parameters that are independent of time. The former must be scaled appropriately for the particular temporal granularity used in the model execution. Ideally, the exact temporal resolution should not fundamentally change the predictions of the model although a too coarse temporal resolution could obviously have detrimental effects and lead to numerical instability (cf. Lomp et al., 2016).

For pure simulation models, it can sometimes be beneficial to adapt the temporal resolution on-line to the necessary precision are each time point. This is especially the case for models expressed in terms of differential equations. However, for models controlling robots, this is usually not practical and a fixed temporal resolution is often more suitable (Balkenius et al., 2020) or a method for aligning simulation time with real-time (Lomp et al., 2016).

## 5 Validating models

To validate the performance of a brain model, there are essentially three complementary ways to do it: structural similarity, functional similarity and behavioral validation.

**Structural similarity** describes the degree to which different brain regions or circuits have similar anatomical connectivity, that is, has similar topology to the real brain. A system-level brain model can vary in terms of its level of structural similarity to the real brain depending on at what resolution the model is specified as discussed above.

For a system-level model, it useful to relate the connections between components to the human or animal connectome to the extent that it is known (Sporns et al., 2005). Automatic methods can be used to make a graph comparison between the model and the relevant aspects of the connectome (Osmanlıoğlu et al., 2019; Pedigo et al., 2023). This assumes that the mapping between components and brain regions is specified in a standardized way. Structural validation can be more feasible in animals with a smaller nervous systems, such as insects that still have a large and complex behavioral repertoire (Goulard et al., 2021). Creatures where the complete neural structure is known such as molluscs (Kandel and Schwartz, 1982) and nematodes (White et al., 1986) also offer this advantage, but simultaneously show very limited behavior compared to more advanced species.

**Functional similarity** refers to the degree these regions or circuits exhibit similar neural activation as seen in the real brain. To validate the functional output of the components that make up a system-level brain model can be tricky for several reasons. First, the notion of functional brain regions is somewhat controversial in the first place (Pang et al., 2023), and second, it can be hard to determine what constitutes the “output” of a neural population, or an area of the brain given its highly recurrent connections. Nevertheless, given some simplifying assumptions such as that the activity of neurons reflect what they are sensitive to, we can infer some transformations that various brain areas appear to do. For example, there appears to be a process of composing larger percepts from smaller building blocks in both the visual- and auditory cortices (Hubel and Wiesel, 1968; Roe et al., 1992). Similarly, winner-takes-all mechanisms appear to mediate decisions and choices involving behavior in the basal ganglia (Berns and Sejnowski, 1996). Another large-scale pattern appears to be that the brain makes extensive use of opponent processing, e.g. between the default mode network and external sensory and processing networks (Smallwood et al., 2012). Common examples of such opponent processes in terms of behavior is approach vs avoidance (Palminteri and Pessiglione, 2017), or “tend-befriend vs. fight-flight” in stressful situations (Turton and Campbell, 2005).

**Behavioral validation** one important function of the brain of animals is to produce behavior that contributes to keeping the animal alive. In contrast to statistical models like linear regression that are validated on how well they can predict data, models of the brain or of parts of the brain can also be partially validated by being able to produce similar behavior to that of an animal, including humans. This is what we mean by “behavioral validation:” if a model can produce functional, embodied behavior, like grasping, manipulation, or navigation that model can tell us something useful. Even if it is clear that the model is doing it in a very simplified way compared to its biological counterpart, it can contribute to our understanding of the biological system by making what is possible concrete.

Behavioral validation typically uses the results of empirical studies as benchmarks. For example, the recorded behavior of an animal during a conditioning experiment is compared to the output of the model using some suitable metric. Different experimental conditions are tested with the model and compared to empirical data.

System-level brain models allows this to be done in a systematic way to evaluate the performance of a model on a large range of experimental set-ups and conditions. Both quantitative and qualitative measures can be used in the behavioral validation. A particularly elegant early example of a model that combines a system-level model different types of qualitative and quantitative validation was presented by Schmajuk and DiCarlo (1992). The model aims at investigating the role of the hippocampus in classical conditioning and the model is tested on a large number of experimental paradigms.

Another more recent example is the model illustrated in Johansson et al. (2020) that aims at reproducing a number of phenomena in the control of pupil dilation with a system-level brain model (Johansson and Balkenius, 2018). This model stands out in that it has almost no parameters. Instead, the anatomical structure of the model is responsible for all qualitative results and surprisingly also the quantitative relation between the time needed for dilation and contraction of the pupil. The model has been implemented in a humanoid robot where it controls its pupils (Johansson et al., 2020). Although the model includes many areas of the brain, it is limited to explaining a very small part of the overall behavior.

In addition to testing individual models, it is also possible to evaluate the behavioral output of a model on a population level by running it multiple times with different parameters selected from a suitable distribution. This is useful when the available empirical data is only reported on a population level or there is a random factor in the recorded output.

To be able to run large-scale simulation is very useful to test models, but to validate that they are able to interact with the real world it is necessary to confront the model with the physical environment. This is best done using robots that are controlled by the model. As stated by Brooks (1990), “the world is its own best model.” By using a physical robot rather than a simulated environment, a model is tested in a more realistic setting. Some of the earliest attempts to use robot to test brain models were published in the 1990s. For example, Edelman and coworkers explored the use of a robot to test the theory of neuronal group selection (Edelman et al., 1992).

Despite its inherent limitations, this marriage between brain models and robots brings some extremely valuable benefits.

First, it makes the model *embodied*. This is not only useful for testing a model but also for shifting focus during development from internal processes to the role they serve in controlling the body (Chiel and Beer, 1997). It also acknowledges that some of the processing is made by the body itself (Pfeifer and Bongard, 2006).

Second, it makes the model *embedded* and *situated* in the environment (Balkenius et al., 2023). It provides input to the networks from the physical world. This is important because the physical world provides rich structure that is hard to replicate in simulations: texture, mass, colors, light and shade, space to move.

Third, a physical robot interacting with the world automatically frames the environment in terms of *affordances* (Balkenius et al., 2023; Gibson, 1977; Cisek and Kalaska, 2010), or which interactions are made possible given a specific robot and a specific network and neuron implementation. The notion of affordance can here be interpreted as opportunities for action that the environment and objects in it allow—in a robot's case it could be *grippability* for an object like a cup, or *navigability* of a doorway or a corridor. In other words, affordances are most easily thought of in terms of manipulations such as grasping, pulling, and shoving. But a robot without arms and hands still has affordances as long as it can move at all: bumping into things is still interaction, albeit a crude one. Notably, affordances are not only dependent on the physical realization of the robot, but also on its control system. Even with a very human-like hand, a robot won't be able to grasp a cup without appropriately sophisticated control software.

Further, the networks that control movement and perception contribute significantly to how a robot might interact with its environment. Sharper perception affords better discrimination; support for salience affords filtering out the things that are important to the task at hand; memory and learning affords learning from trial and error, but can also afford simulation of action and consequence, and navigating environments based on experience. In short, the neurorobotics approach provides opportunities to study cognition as a natural phenomenon that is closer to “cognition in the wild” than is simulation of neural networks, and it provides a degree of control over experiments that is superior to behavioral experiments on animals. By grounding brain models in robotic experiments, and keeping the demands of a biological organism in focus makes for more realistic models that have a greater potential to explain how the brain interacts with the body and the environment (Krichmar and Hwu, 2022). Aiming at models that will eventually control a robot structures the modeling work toward a system that can operate in the real world. This approach supports both computational rigor and the generation of realistic behavioral outputs.

Since robots are not exact copies of humans (or animals), it will not be possible in most cases to replicate the exact behavior of a human. Instead, the validation of system-level brain models in robots must also include qualitative aspects of behavior. For example, it is not meaningful to try to replicate the exact reaction times in a task since it will depend on the physical components of the robot rather than the details of the model. It is however interesting to look at the change in reaction time over time or how a motor action changes as a result of training.

**Figure 2:**
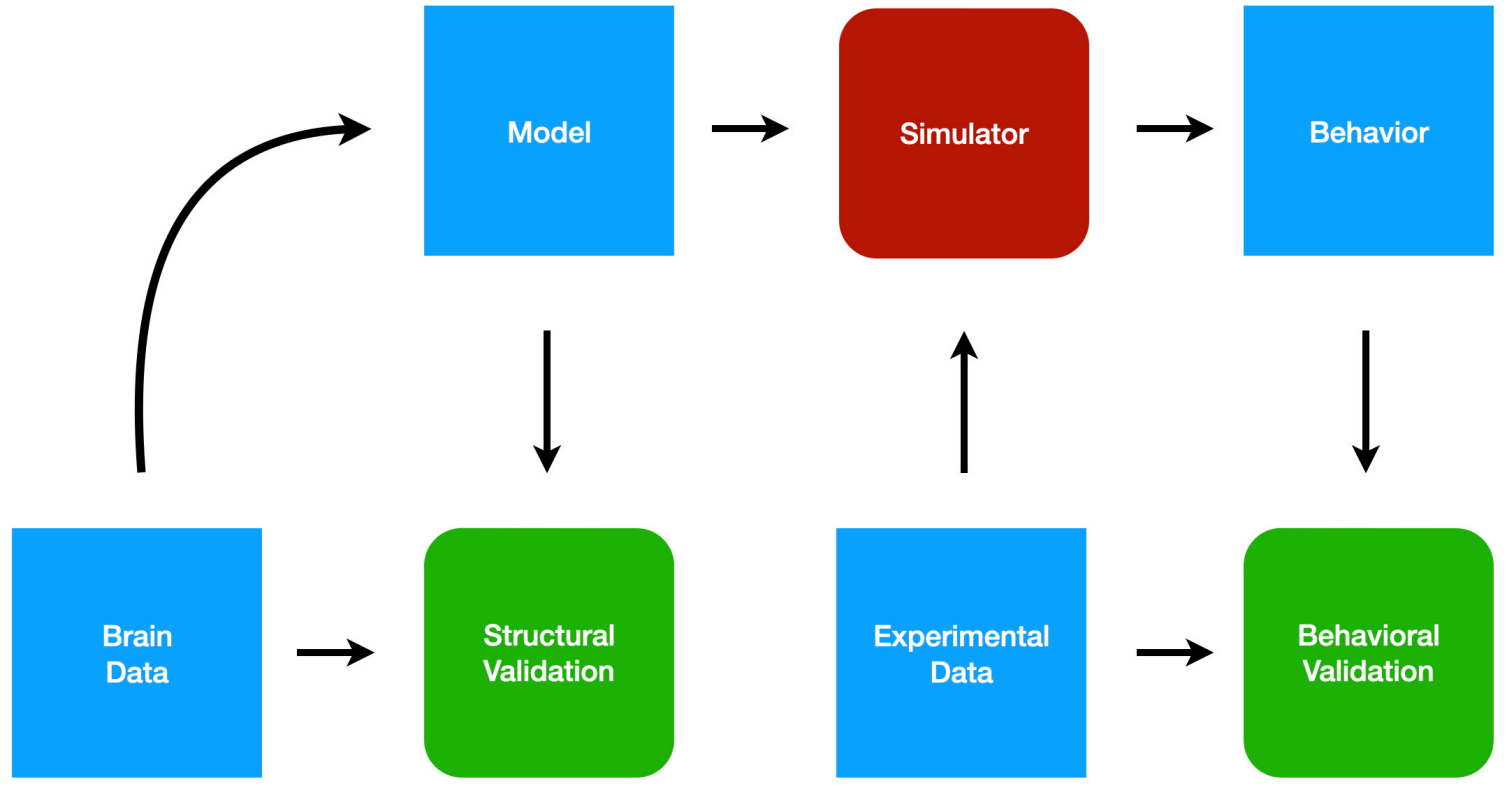
The modeling pipeline.

Figure 2 shows the simulation pipeline. Here we describe the Ikaros system, but the methodology is relevant also to other simulation approaches. Starting with brain data in the form of the connectome and other relevant data, such as receptor types, a formal mathematical model is created. The model is described in an XML-based language that allows the specification of the components and their interaction as well as the hierarchical organization of the model (Balkenius et al., 2010, 2020). The formal model description can subsequently be structurally validated by comparing it to the brain data that was used to create it. The next step is to test the model using a number of data sets that probes the functionality of the model. The experimental data is used first to set up the experimental conditions for each simulation and second to validate the behavioral output from the simulations.

## 6 A case study: control of pupil dilation

**Figure 3:**
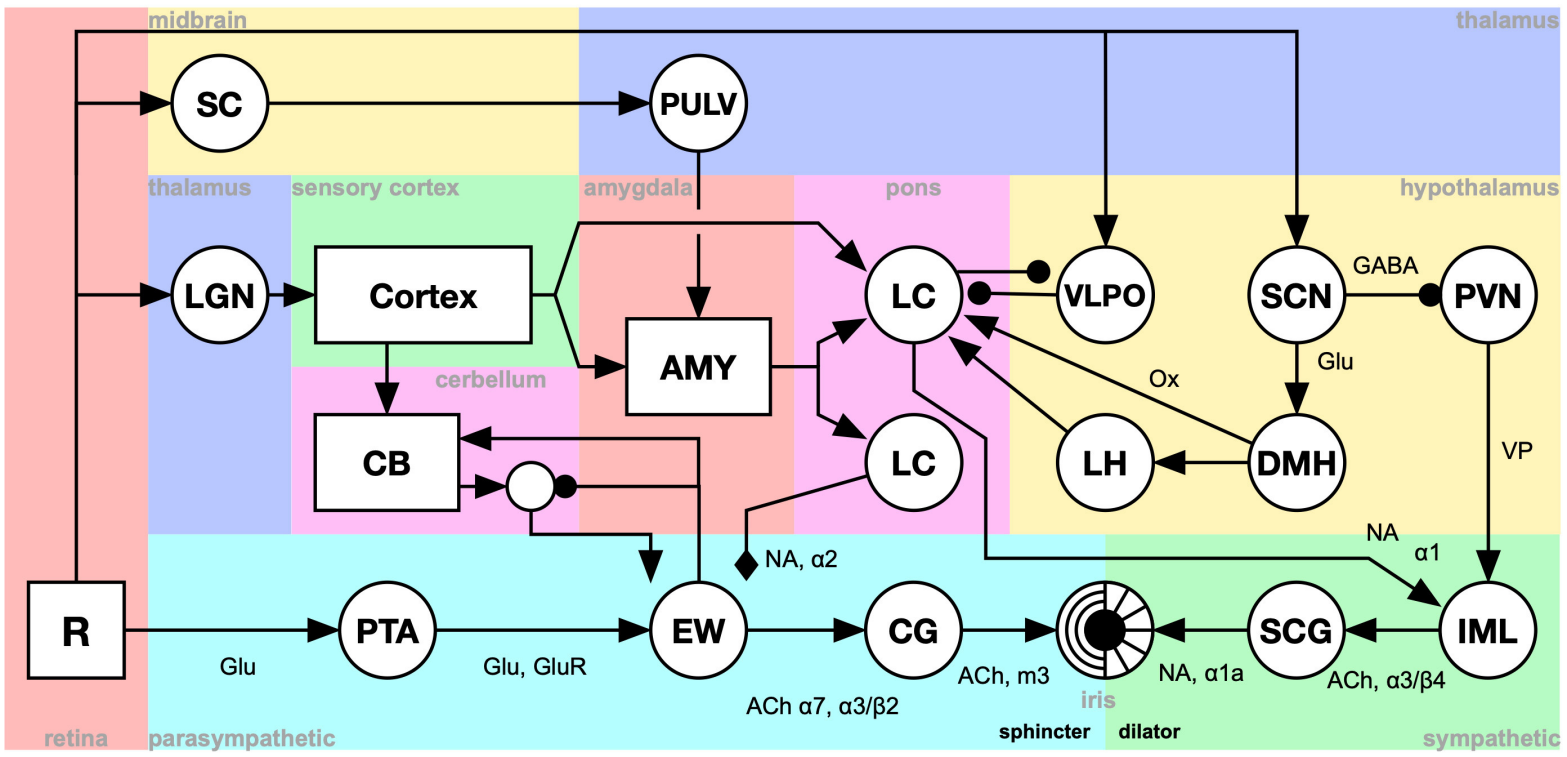
A system-level model of pupil dilation. R, The retina; PTA, Pretegmental area; EWpg, Edinger-Westphal nucleus, preganglion portion; CG, ciliar gangion; SCG, superior cervical ganglion; IML, intermedio-lateral column of the spinal cord; CB, cerebellum; LH, lateral hypothalamus; DMH, dorsomedial hypothalamus; VLPO, ventrolateral preoptic nucleus; SCN, suprachiasmatic nucleus; PVN, paraventricular nucleus; LC, locus coeruleus; AMY, amygdala; LGN, lateral geniculate nucleus; SC, superior colliculus; PULV, the pulvinar of the thalamus [Adapted from Johansson and Balkenius (2018), Balkenius et al. (2019) and Tjøstheim et al. (2019)].

In this section, we present an example of a system-level brain model of pupil control. Pupil dilation provides a rich testbed for modeling the integration of cognitive, emotional, and sensory inputs. The model puts the concepts introduced above in context and illustrates a number of aspects of system-level models (Figure 3).

Most parts of the model are implemented using a single nucleus-model indicated by the circular elements of the figure. This level of detail is sufficient to capture the dynamics of the nuclei involved in pupil control. Each nucleus is modeled using the following equation, where *E*_*i*_ are the excitatory inputs, *I*_*i*_ are direct inhibitory inputs and *S* is shunting inhibition. This captures the dynamics of the different receptor types that are relevant to the model as show in the figure.


(1) $$\begin{array}{l} \epsilon \frac{dx}{dt}=\alpha +\beta \left(\frac{1}{1+S}\right)\overset{N}{\underset{i=1}{\sum }}E_{i}w_{i}u-\gamma \overset{M}{\underset{j=1}{\sum }}I_{j}-x \end{array}$$

The resting level is defined by α, while β and γ scale excitation and inhibition, respectively. Unless specified otherwise, they are set to average the inputs: β = 1/*N*, γ = 1/*M*. This makes the processing of the model stable as more connections are added since the total input will always stay in the range [0, 1].

Excitatory weights *w*_*i*_ are fixed at 1 for all non-plastic synapses, except in the amygdala and cerebellum. Each synapse has an input gain *u*, typically 1, which slowly adapts to the average input, modeling receptor up- and downregulation. This adaptation occurs on a time scale slower than the behavioral simulations and represents a dynamic gain control mechanism. The nucleus output is given by *o* = ϕatan(*x*), with ϕ = 1/atan(1) to ensure that an input of 1 yields an output of the same magnitude, preventing signal amplification at this stage.

Other components have more complex structure and are illustrated by rectangles. This includes the visual cortex, the cerebellum (CB) and the amygdala (AMY) (See Johansson and Balkenius, 2018 for further details).

The overall structure of the model is given by the connection graph, as illustrated by arrows in the figure. The type of arrow also indicates the type of receptor dynamics used in each case.

The coding of the signals in the model is mainly as the scalar output of the nuclei components. However, the rectangular components in the figure use different types of matrix coding. For example, the retina (R) uses a matrix to represent a gray scale image while the cortex output is a single array coding the identity of a recognized stimuli used by the cerebellum component as well as surprise signals sent to the amygdala.

Timing is modeled using a delay in the signal path between each pair of components. This delay is equal for all components except for the cerebellum where it was adjusted to capture the optimal inter-stimulus interval for classical conditioning.

The full model runs in real time on standard consumer hardware, such as an Apple Mac Mini, without specialized processing hardware. This means that users do not need specialized or high-end hardware to execute the model effectively.

The model was validated both in simulation in a humanoid robot and reported in three papers that collectively reproduce a broad range of experimental phenomena related to pupil dynamics, trust formation, emotion, and cognition, using system-level simulations. This model is evaluated through simulations that qualitatively and quantitatively mirror empirical findings.

In the study by Johansson and Balkenius (2018), we presented a model that successfully reproduced five key phenomena associated with pupil dynamics. First, it replicated the *pupillary light reflex*, including the characteristic shape of the response curve and the latency and magnitude scaling with stimulus intensity, consistent with Ellis (1981). Second, the model elicited *pupil dilation in response to novel stimuli*, showing greater dilation during first exposures compared to subsequent ones—a qualitative result supported by empirical findings (Aboyoun and Dabbs, 1998). Third, the model demonstrated *emotional reactivity*, with stronger pupil dilation to emotionally charged stimuli regardless of polarity (positive or negative), matching the findings of Hess and Polt (1960). Fourth, it reproduced conditioned responses to *images associated with brightness*, such as the sun, even when actual luminance was equal, simulating the *learned “light response”* via the cerebellum; this is a qualitative result tightly linked to the experimental design by Binda et al. (2013). Lastly, the model captured the *fear-inhibited light reflex*, wherein the pupil constriction in response to light was reduced when preceded by a fear-conditioned stimulus—matching experimental findings from Bitsios et al. (1996).

Building on this, Balkenius et al. (2019) extended the model to include additional structures such as the superior colliculus and the pulvinar, enabling it to simulate *pupillary contagion*—a phenomenon where an observer's pupils dilate in response to seeing dilated pupils in others. The model reproduced empirical results from infant studies and adult visual perception by showing greater dilation to larger circular stimuli simulating eyes, but no response to squares, thus offering a quantitative match to data reported by Fawcett et al. (2017). It also replicated *cognitive effort effects*, where pupil dilation increased with the difficulty of arithmetic problems. Using simulated training and emotionally conditioned associations, the model generated a dilation curve closely mirroring empirical data from Kahneman and Beatty (1966) and Hess and Polt (1964). Furthermore, the model simulated various *hypotheses about pupil anomalies in autism spectrum disorder*, such as increased baseline dilation due to sympathetic tone or increased light reflex magnitude from α7-receptor upregulation. These results were partly quantitative (e.g., changes in latency and amplitude) and partly qualitative, aligning with observations from studies like Nyström et al. (2015) and Anderson and Colombo (2009).

Lastly, Tjøstheim et al. (2019) extended the model further to incorporate *trust, motivation, and tactile feedback* to model *unconditional trust formation* based on familiarity and gentle or painful touch stimuli. The model showed that trust increased with familiarity and gentle touch, while painful touch significantly reduced trust and increased avoidance motivation. These changes were accompanied by corresponding shifts in pupil dilation, with pain and distrust correlating with higher dilation—findings that aligned with empirical results by Kret et al. (2015) and Höfle et al. (2008). The results were primarily quantitative, as the model produced explicit numerical values for trust, approach/avoidance motivation, and pupil dilation. Additionally, when faces were reintroduced without tactile input, the model's trust responses reflected prior associations, demonstrating a form of *context-dependent memory effect* in pupil and motivational dynamics—a result that is both qualitative and quantitative.

Together, these studies demonstrate that system-level, biologically plausible models can reproduce a wide variety of pupil-linked cognitive and emotional effects observed in humans, providing both mechanistic insight and computational tools for future research in affective neuroscience and human-agent interaction.

**Figure 4:**
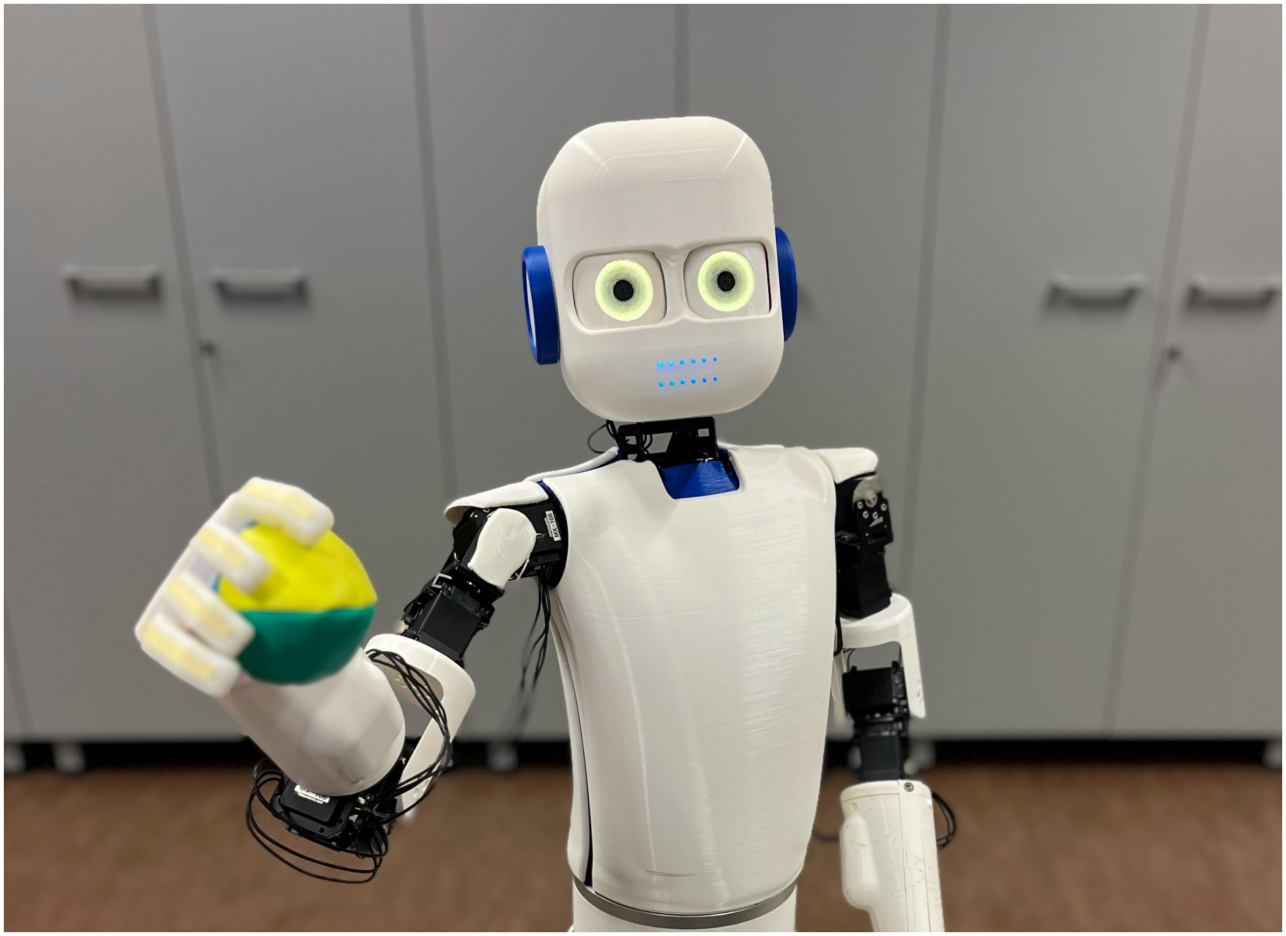
The Epi robot used for system-level brain model of pupil control.

For the robotics validation, we used the Epi robot (Figure 4) (Johansson et al., 2020). Only minimal changes were made to the model. The component implementing the visual cortex was replaced with a model that could categorize objects from the visual input from the two cameras in the eyes of the robot. In addition, the delayed connection used to model the optimal inter-stimulus interval for conditioning was replaced by a tapped delay-line to accommodate a larger variability in the timing in this real-world situation compared to the simulation. Furthermore, the output was sent to the servo controllers for the pupils of the robot. This allowed the different experiment that was initially tested in simulation to be run on the robot. Here, the real-time features of the Ikaros framework were essential.

## 7 Discussion

We have argued that system-level brain modeling is a viable approach to developing and testing brain models. The main advantage of system-level models is that it allow a level of abstraction that makes large-scale brain models within reach also with modest computational resources. This contrasts with models specified at a lower level that may require high-performance computing resources.

A central question to ask when using models to understand how brains and nervous systems work is: *What is a good model?* The validity of the model depends, of course, on what we are trying to understand, and thus how well the particular model confers the insights we are seeking. But given that we are in fact wanting to understand the whole brain, or even the entire cognitive system, including the interactions with its body and its environment, it is clear that the model must be both embodied and situated. That is, we need a physical robot that can engage with its environment, and which is controlled by a model that has the necessary analogies to a biological central nervous system.

A future direction for the field is to develop benchmarking standards for brain models comparable to those used in artificial neural networks. This makes it easy to see if progress has been made. First, for the model to generate insights about the brain as a whole, it should consist of an analogous number of systems as does the biological brain, and the scope of these systems should map to their biological counterparts as well. The challenge then is to find the appropriate resolution of the model and of its components. Second, the model should display computational soundness. Here it is useful to recall Marr's levels of analysis regarding computation: at the highest level is the outcome of a process, called the computational level; next down is the algorithmic level concerning how the computation is done using available primitives; and lowest is the implementation level, concerning how primitives are realized.

A more straightforward way of looking at the problem is to simply consider whether the model solves the problem under scrutiny at all like a biological brain. From a neurobiological perspective, models that rely heavily on iterative computational structures typical of conventional computer science may not align well with how biological systems process information. On the other hand, if the solution depends instead on excitation, inhibition, and modulation, this may suggest a closer alignment with biological plausibility.

Third, the number of behavioral or other experimental paradigms that can be reproduced qualitatively and quantitatively should be maximized. This implies that the same model should be able to generate behaviors sufficiently rich that could be applied to a wide set of experimental paradigms from behavioral psychology. Examples thus include executive paradigms such as the Flanker task (Eriksen and Eriksen, 1974) where the experimental participant is required to inhibit distractor stimuli that are either congruent or incongruent with the centrally positioned stimuli, as well as reaction-time tasks such as go-no go paradigms where participants must react only to stimuli that are occasionally presented, while inhibiting behavior most of the time. In practice though, the model should display a wide range of cognitive processing including attention, perception, executive function, and memory, all of which are amendable to testing with standard protocols.

Fourth, given that the above experiments can be simulated using the model, the output should quantitatively match results reported in the literature. This requirement puts rather tight constraints on the real-time performance of the model, entailing in effect millisecond resolution on its turnaround or step time. In addition to appropriate responses with realistic temporal signatures, the model should also reproduce measures like EEG-like recording and activation of regions analogous to those recorded with fMRI techniques. If this can be achieved, the model would allow for broad experimentation to tease out functional relationships in the brain, and should be able to produce robust predictions for validation in animal- or human experiments.

To date, there have been few attempts to systematically test system-level brain models in robots on a large range of behavioral data from empirical research. As a concrete step toward this vision, we are developing the BAM model of the brain. The model runs on the Ikaros framework and is currently used to control the humanoid robot Epi. When complete, the model will include several hundred brain regions and be tested on a wide range of empirical data. The goal is to be able to perform basic neurological tests on the robot as well as to test it on a large set of experimental paradigms including classical and instrumental conditioning, visual attention and memory tasks, eye-hand coordination, and spatial navigation. Many of the required components have been developed over the last 20 years and are ready to be combined into an integrated system-model. The methodology outlined in this paper offers a structured framework for advancing the integration of system-level modeling with empirical validation.

## Data Availability

The original contributions presented in the study are included in the article/supplementary material, further inquiries can be directed to the corresponding author.
